# Influence of neutropenia on mortality of critically ill cancer patients: results of a meta-analysis on individual data

**DOI:** 10.1186/s13054-018-2076-z

**Published:** 2018-12-04

**Authors:** Quentin Georges, Elie Azoulay, Djamel Mokart, Marcio Soares, Kyeongman Jeon, Sandra Oeyen, Chin Kook Rhee, Pascale Gruber, Marlies Ostermann, Quentin A. Hill, Pieter Depuydt, Christelle Ferra, Anne-Claire Toffart, Peter Schellongowski, Alice Müller, Virginie Lemiale, Fabien Tinquaut, Aurélie Bourmaud, Michaël Darmon

**Affiliations:** 1 Medical-Surgical ICU, Saint-Etienne University Hospital, Avenue Albert Raimond, 42270 Saint-Priest-en-Jarez, France; 2 Jacques Lisfranc Saint-Etienne Medical School, Jean Monnet University, Saint-Priest-en-Jarez, France; 3 Medical ICU, Saint-Louis University Hospital, AP-HP, Paris, France; 4 Sorbonne-Paris-Cité, Medical School, Paris-Diderot University, Paris, France; 5 Anesthesiology and Intensive Care Unit, Institut Paoli Calmette, Marseille, France; 6 Department of Critical Care and Graduate Program in Translational Medicine, D’Or Institute for Research and Education, Rio de Janeiro, Brazil; 7 Department of Critical Care Medicine, Samsung Medical Center, Sungkyunkwan University School of Medicine, Seoul, South Korea; 8 Department of Intensive Care Medicine, Ghent University Hospital, Ghent, Belgium; 9 Division of Pulmonary and Critical Care Medicine, Department of Internal Medicine, The Catholic University of Korea, Seoul, Korea; 10 Department of Critical Care, The Royal Marsden Hospital, Fulham Road, London, SW3 6JJ UK; 11 Department of Critical Care & Nephrology, Guy’s & St Thomas’ NHS Foundation Hospital, Westminster Bridge Road, London, UK; 12 Department of Haematology, Leeds Teaching Hospitals, Leeds, UK; 13 Department of Clinical Hematology, ICO-Hospital Germans Trias i Pujol, Josep Carreras Research Institute, Universitat Autònoma de Barcelona, Badalona, Spain; 14 Thoracic Oncology Unit, Grenoble Alpes University Hospital, Grenoble, France; 15 Department of Medicine I, Intensive Care Unit 13i2, Comprehensive Cancer Center, Medical University of Vienna, Vienna, Austria; 16 Universidade Federal do Rio Grande do Sul, Porto Alegre, Rio Grande do Sul Brazil; 17 Hygée Centre and Public Health Department, Lucien Neuwirth Cancerology Institute, Saint-Priest-en-Jarez, France; 18 INSERM, ECSTRRA team, UMR 1153, Paris, France

**Keywords:** Prognosis, Outcomes, Hematologic, Neoplasms, Intensive care units, Mechanical ventilation, Neutropenia

## Abstract

**Background:**

The study objective was to assess the influence of neutropenia on outcome of critically ill cancer patients by meta-analysis of individual data. Secondary objectives were to assess the influence of neutropenia on outcome of critically ill patients in prespecified subgroups (according to underlying tumor, period of admission, need for mechanical ventilation and use of granulocyte colony stimulating factor (G-CSF)).

**Methods:**

Data sources were PubMed and the Cochrane database. Study selection included articles focusing on critically ill cancer patients published in English and studies in humans from May 2005 to May 2015. For study selection, the study eligibility was assessed by two investigators. Individual data from selected studies were obtained from corresponding authors.

**Results:**

Overall, 114 studies were identified and authors of 30 studies (26.3% of selected studies) agreed to participate in this study. Of the 7515 included patients, three were excluded due to a missing major variable (neutropenia or mortality) leading to analysis of 7512 patients, including 1702 neutropenic patients (22.6%). After adjustment for confounders, and taking study effect into account, neutropenia was independently associated with mortality (OR 1.41; 95% CI 1.23–1.62; *P* = 0.03). When analyzed separately, neither admission period, underlying malignancy nor need for mechanical ventilation modified the prognostic influence of neutropenia on outcome. However, among patients for whom data on G-CSF administration were available (*n* = 1949; 25.9%), neutropenia was no longer associated with outcome in patients receiving G-CSF (OR 1.03; 95% CI 0.70–1.51; *P* = 0.90).

**Conclusion:**

Among 7512 critically ill cancer patients included in this systematic review, neutropenia was independently associated with poor outcome despite a meaningful survival. Neutropenia was no longer significantly associated with outcome in patients treated by G-CSF, which may suggest a beneficial effect of G-CSF in neutropenic critically ill cancer patients.

**Systematic review registration:**

PROSPERO CRD42015026347. Date of registration: Sept 18 2015

**Electronic supplementary material:**

The online version of this article (10.1186/s13054-018-2076-z) contains supplementary material, which is available to authorized users.

## Background

Cancer is a leading cause of death in North America and Europe [1, 2], and cancer patients are at high risk for life-threatening complications as a result of infection [3], toxicity of intensive treatments [4] or targeted therapies [5], warranting admission to the intensive care unit (ICU). Despite evidence that ICU mortality rates have declined significantly over the last two decades [6, 7], and although the number and extent of comorbidities, pre-existing performance status along with organ failure have been demonstrated to be the main prognostic factors in this setting [8–10], intensivists may be reluctant to admit specific cancer patient populations such as neutropenic patients.

Although meaningful survival has been described in neutropenic patients [11, 12], the prognostic impact of neutropenia remains controversial. Neutropenia remains a common side effect of cancer chemotherapy and, although transient, may lead to immune dysfunction. Clinical consequences of neutropenia are well known and include occurrence of sepsis or acute respiratory failure [13], worsening of respiratory status during neutropenia recovery [14] and need for specific management [15]. In contrast to critically ill cancer patients, neutropenia was found to be an independent risk factor of poor outcome in the general ICU population [16]. The lack of prognostic impact in critically ill cancer patients may thus reflect an absence of statistical power or the influence of coexistent mechanisms of immune deficiency in these patients. In a previous systematic review performed on aggregated data, neutropenia was associated with an increase in relative risk of death of 10% in critically ill cancer patients [17]. With regards to the limited number of studies reporting an adjusted impact of neutropenia, however, this preliminary study failed to demonstrate an independent impact of neutropenia on outcome [17].

The aim of this study was to assess influence of neutropenia on outcome of critically ill cancer patients by a meta-analysis of individual data. Secondary objectives were to assess influence of neutropenia on outcome of critically ill patients in prespecified subgroups (according to underlying tumor, period of admission, need for mechanical ventilation and use of granulocyte colony stimulating factor (G-CSF)).

## Methods

This systematic review and meta-analysis of individual data was performed according to the guidelines on Meta-analysis of Observational Studies in Epidemiology [18]. This study was registered on the PROSPERO database (CRD42015026347). This study was a preplanned follow-up study of an initial meta-analysis on aggregated data [17].

### Study outcome and definitions

The aim of this meta-analysis of individual data was to determine the prognostic impact of neutropenia on outcome of critically ill cancer patients.

Neutropenia was defined as a neutrophil count (or if missing as a white blood cell count) lower than 1 G/L (stage 3 or more according to Common Terminology Criteria for Adverse Events version 4.03) (https://evs.nci.nih.gov/ftp1/CTCAE/CTCAE_4.03/CTCAE_4.03_2010-06-14_QuickReference_5x7.pdf).

Outcome was defined as hospital mortality or day-28 mortality if the former was unavailable (Table 1). Choice of this outcome variable was driven by availability of data precluding use of longer follow-up such as day 90. Although ICU mortality was available for most of the reported studies, this variable is more prone to be influenced by the discharge policy in participating centers and a longer follow-up period was preferred [19].

**Table 1:** Included studies’ characteristics

Author, year	Follow-up	*n*	SAPS II equivalent	Prospective study	Number of centers	Solid tumors (%)	Mortality (%)	Risk of bias
Canet et al., 2013 [34]	Hospital	200	34 (24–50)	Yes	1	0	30.0	7
Depuydt et al., 2010 [33]	Hospital	137	25 (20–30)	No	1	2.2	62.8	7
Bird et al., 2012 [27]	Hospital	199	21 (17–25)	Yes	1	0	54.3	5
Hill et al., 2012 [28]	Hospital	147	62 (48–80)	No	5	0	73.5	5
Müller et al., 2013 [35]	Hospital	34	57 (31–97.75)	No	1	100	44.1	5
Namendys-Silva et al., 2012 [30]	Hospital	184	40 (31–51)	Yes	1	30.4	58.2	6
Yeo et al., 2012 [48]	Hospital	227	54 (37–71)	No	1	0	89.9	7
Oeyen et al., 2013 [36]	Hospital	483	34 (22–48)	Yes	1	82.4	16.4	6
Ferra et al., 2007 [37]	Hospital	100	60 (44.25–71)	Yes	1	0	64.0	5
Soares et al., 2006 [20]	Hospital	309	56 (45–68)	Yes	1	24.6	64.1	7
Soares et al., 2007 (*Chest*) [38]	Hospital	143	44 (32.5–57)	Yes	2	100	58.7	6
Soares et al., 2007 (*Intensive Care Med*) [44]	Hospital	121	47 (37–62)	Yes	1	0	56.2	6
Soares et al., 2008 [32]	Hospital	1090	44 (32–56.75)	Yes	1	81.8	51.2	7
Soares et al., 2010 [25]	Hospital	717	29 (19–42)	Yes	28	93	30.4	7
Souza-Dantas et al., 2011 [45]	Hospital	188	62 (48.75–73)	No	1	31.9	75.5	7
Soares et al., 2014 (46)	Hospital	449	44 (33–55)	Yes	22	100	38.8	5
Song et al., 2011 [26]	Hospital	62	41 (25–51)	No	1	21	69.4	6
Yoo et al., 2013 [42]	Hospital	214	44 (35–59)	No	1	46.3	49.1	7
Lee et al., 2015 [43]	Hospital	525	61.5 (51–70)	Yes	1	40.2	56.0	7
Mokart et al., 2007 [21]	28 days	51	49 (35.5–70.25)	Yes	1	21.6	43.1	5
Mokart et al., 2012 [47]	Hospital	111	45 (33–55)	Yes	1	21.6	40.5	6
Adda et al., 2008 [41]	Hospital	99	49 (39.5–57)	No	1	0	61.6	6
Burghi et al., 2011 [40]	28 days	59	NA	No	1	0	72.9	7
Legriel et al., 2010 [23]	Hospital	101	55 (42–67)	No	1	29.7	44.6	5
Xhaard et al., 2013 [29]	Hospital	62	NA	No	1	0	41.9	5
Azoulay et al., 2008 [22]	Hospital	148	NA	Yes	1	12.8	55.4	6
Azoulay et al., 2013 [8]	28 days	1011	29 (23–39)	Yes	17	0	38.2	7
McGrath et al., 2010 [24]	Hospital	185	39 (26–47)	No	1	37.8	31.4	5
Wohlfarth et al., 2014 [31]	Hospital	56	50 (39–60.5)	No	1	14.3	41.1	5
Toffart et al., 2011) [39]	Hospital	103	44 (33–57)	No	3	100	31.1	7

*SAPS* Simplified Acute Physiology Score

### Search strategy and eligibility assessment

First, public-domain databases including PubMed and the Cochrane database were searched using Exploded Medical Subject Headings and the appropriate corresponding keywords: “NEOPLASM” OR “MALIGNANCY” OR “CANCER” AND “INTENSIVE CARE UNIT” OR “ICU”. The research was restricted to publications in English and studies concerning humans from May 2005 to May 2015. Studies published before 2005 not included to limit heterogeneity across studied populations that may have arisen with regard to prognostic change of both critically ill and critically ill cancer patients [20, 21]. Abstracts were carefully checked and studies focusing on children or patients aged younger than 18 years old, case reports and studies failing to focus on critically ill patients were excluded.

All remaining references were then downloaded for consolidation, elimination of duplicates and further analysis. Four investigators (Marie Bouteloup, Sophie Perinel, DM, MD) independently determined the eligibility of all studies identified in the initial research. Last, studies with explicit redundancies were only included once (for this study, redundancies were assessed by QG and MD).

Each of the included studies obtained approval of a local or a national ethic committee in accordance with local legislation. Patients or their next of kin consented to participate or were informed of the included study and did not oppose participation according to local legislation. With regard to its retrospective design and lack of change of the primary outcome variable, the need for additional ethic committee approval was waived according to French Law.

Patients were not involved in the included studies’ design. The primary outcome measure was defined according to its clinical relevance for both patients and carers. Health care providers were involved in patient recruitment. Last, the patients and physicians involved in the analyzed studies are thanked in Acknowledgements.

### Data and quality assessment

Investigators of selected publications were contacted twice and invited to participate in this study. Authors who agreed to participate were asked to send a file containing individual data including: age, gender, year of admission, underlying malignancy (solid tumor vs hematological malignancy), history of allogeneic stem cell transplantation, severity score, need for organ support (invasive mechanical ventilation, vasopressor use, renal replacement therapy), neutropenia during ICU stay, neutropenia duration, use of granulocyte colony stimulating factor (G-CSF), follow-up and outcome.

To enable study comparison, we transformed illness severity scores (Acute Physiology and Chronic Health Evaluation (APACHE) II and APACHE III) into the equivalent Simplified Acute Physiology Score (SAPS) II, using a previously described methodology [22]. When neither the APACHE II score nor the APACHE III score was available, the available severity score was transformed into the SAPS II according to the estimated odds of dying during the ICU stay.

Risk of bias of included studies was assessed using the “risk bias in cohort study” tool developed by the Cochrane group (http://methods.cochrane.org/sites/methods.cochrane.org.bias/files/public/uploads/Tool%20to%20Assess%20Risk%20of%20Bias%20in%20Cohort%20Studies.pdf; accessed February 2, 2017).

### Statistical analysis

All quantitative variables were described using medians (quartiles) while qualitative variables were described with their frequencies (percentage). The overall association between mortality and patient characteristics was determined with a one-step meta-analysis approach. Univariate analyses were performed using a logistic regression model with random study effects to obtain odds ratios (ORs) with their two-sided 95% confidence intervals (CIs) as measures of relative risk. Variables identified as being associated with mortality in univariate analyses, with *P* < 0.20, were included in a multivariate logistic regression model with a random study effect. Chi-square heterogeneity tests were used to test for statistical heterogeneity among studies. The *I*^2^ index (expressing the proportion of variability of the results related to heterogeneity) was reported.

Strata analyses were performed using the statistical plan already described for the following strata: patients with ICU admission after 2007 (median ICU admission period in the studied population), patients with hematological malignancy, patients requiring mechanical ventilation and patients receiving G-CSF.

All effect sizes with *P* < 0.05 were considered significant. All analyses were carried out with software R, version 3.2.5. The lme4 and Meta package were used to take into account the random effects and to produce forest plots.

## Results

**Fig. 1:**
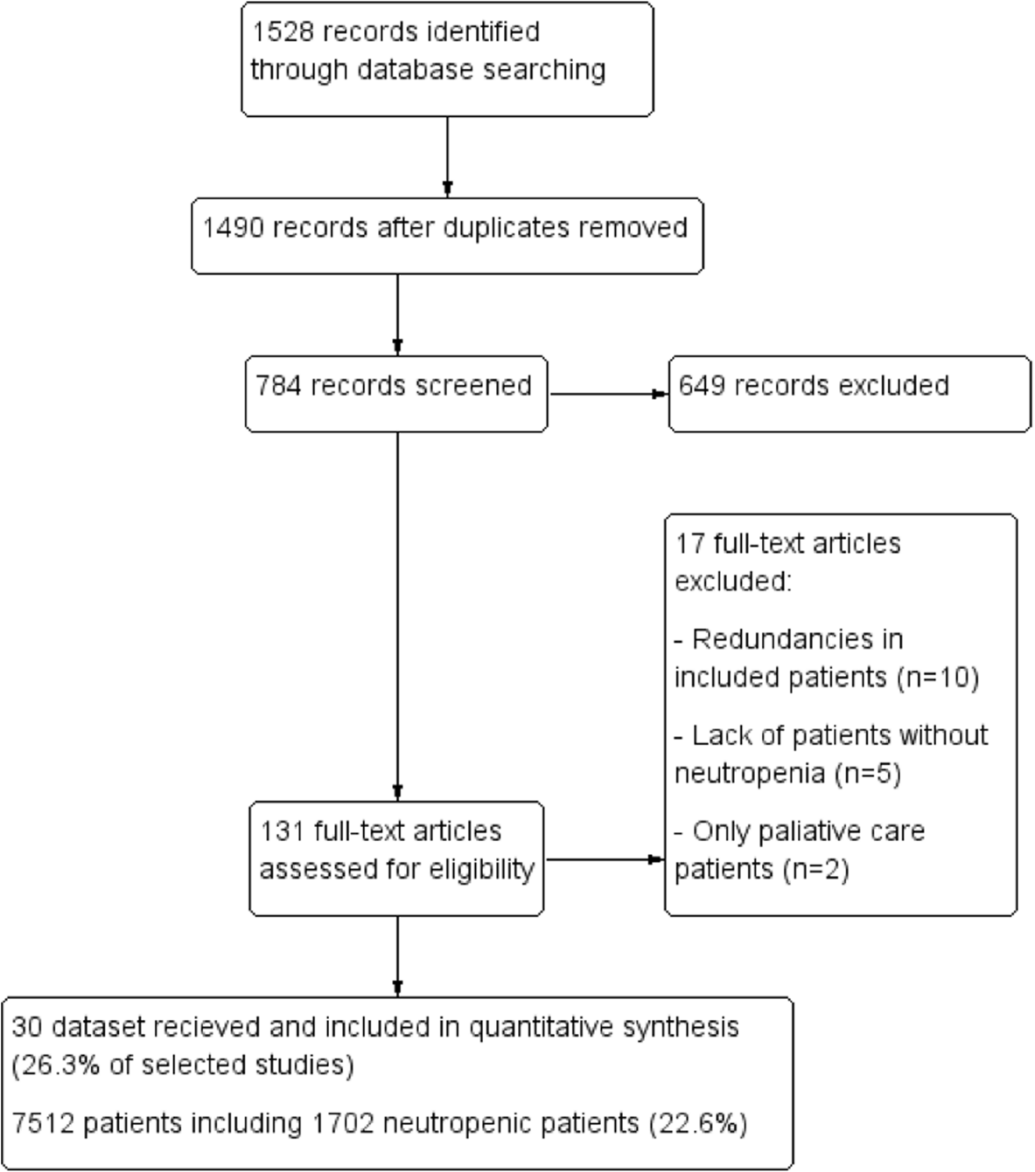
Flow chart of study selection

Our initial search yielded 1528 citations, of which 38 were excluded due to duplication and 706 were excluded as irrelevant for the scope of this review. All abstracts of the remaining 784 records were carefully checked and 131 full-text articles focusing on critically ill cancer patients’ prognosis were scrutinized for further evaluation. Seventeen studies were excluded, including 10 studies with redundancies, five studies lacking neutropenic, non-neutropenic patients or major data required for the analysis, and two studies including only palliative patients. Among the remaining 114 studies, authors of 30 studies (26.3% of selected studies) agreed to participate in this study, leading to a dataset of 7515 patients, including 1702 neutropenic patients (22.6%) (Fig. 1) [8, 23–51].

### Characteristics of included studies

The included studies were published from 2006 to 2015. Sixteen were prospective cohort studies (53%) and six (20%) were multicentric cohort studies (Table 1). The variable of outcome was hospital mortality in 27 studies and 28-day mortality in three studies. Overall, the number of patients included per study was 166 (IQR 99–266) and ranged from 34 [38] to 1090 [35].

### Characteristics of the patients

Of the 7515 included patients, three were excluded due to missing major variables (neutropenia or mortality) leading to analysis of 7512 patients. Of these patients, 4943 were included in monocenter studies (65.8%) and 5841 (77.8%) in prospective studies (Table 2).

Median age was 60 years (49–69) and median SAPS II was 42 (28–57) (Table 2). Overall, 3366 patients (44.8%) had a solid tumor and 439 patients were allogeneic stem cell transplant recipients (5.8%). Respectively, 3710 patients (49.4%), 3084 patients (41.1%) and 1201 patients (16%) required invasive mechanical ventilation, vasopressors or renal replacement therapy.

**Table 2:** Patient characteristics

Variable	Missing data, *n* (%)	Overall population (*n* = 7515)	Neutropenic patients (*n* = 1702)^a^	Non-neutropenic patients (*n* = 5810)^a^	*P* value
Age (years)	15 (0.2%)	60 (49–69)	55 (41–64)	61 (51–70)	< 0.001
ICU admission year	0	2007 (2004–2010)	2008 (2005–2010)	2007 (2004–2010)	< 0.001
Underlying malignancy					
Solid tumors	0 (0%)	3366 (44.8%)	232 (13.6%)	3131 (53.9%)	< 0.001
Hematological malignancy	0 (0%)	4149 (55.2%)	1470 (86.4%)	2679 (46.1%)	< 0.001
Allogeneic HSCT	0 (0%)	439 (5.8%)	186 (10.9%)	253 (4.4%)	< 0.001
SAPS II	503 (6.7%)	41 (28–57)	51 (34–68)	39 (26–54)	< 0.001
Organ support at ICU admission					
Mechanical ventilation	1 (0%)	3804 (50.6%)	964 (56.6%)	2839 (48.9%)	< 0.001
Vasopressors	0 (0%)	3084 (41%)	954 (56.1%)	2129 (36.6%)	< 0.001
Renal replacement therapy	58 (0.8%)	1201 (16%)	386 (22.7%)	815 (14%)	< 0.001
Mortality	2 (0%)	3538 (47.1%)	1021 (60%)	2517 (43.3%)	< 0.001

*HSCT* hematopoietic stem cell transplantation, *SAPS* Simplified Acute Physiology Score, *ICU* intensive care unit

^a^Data on neutropenia unavailable in three patients (0.03%)

### Outcome

Unadjusted mortality in the studied population was 47.1% (*n* = 3538), including mortality of 60.2% (*n* = 1025) and 43.2% (*n* = 2504) in neutropenic and non-neutropenic patients, respectively (*P* < 0.001).

**Fig. 2:**
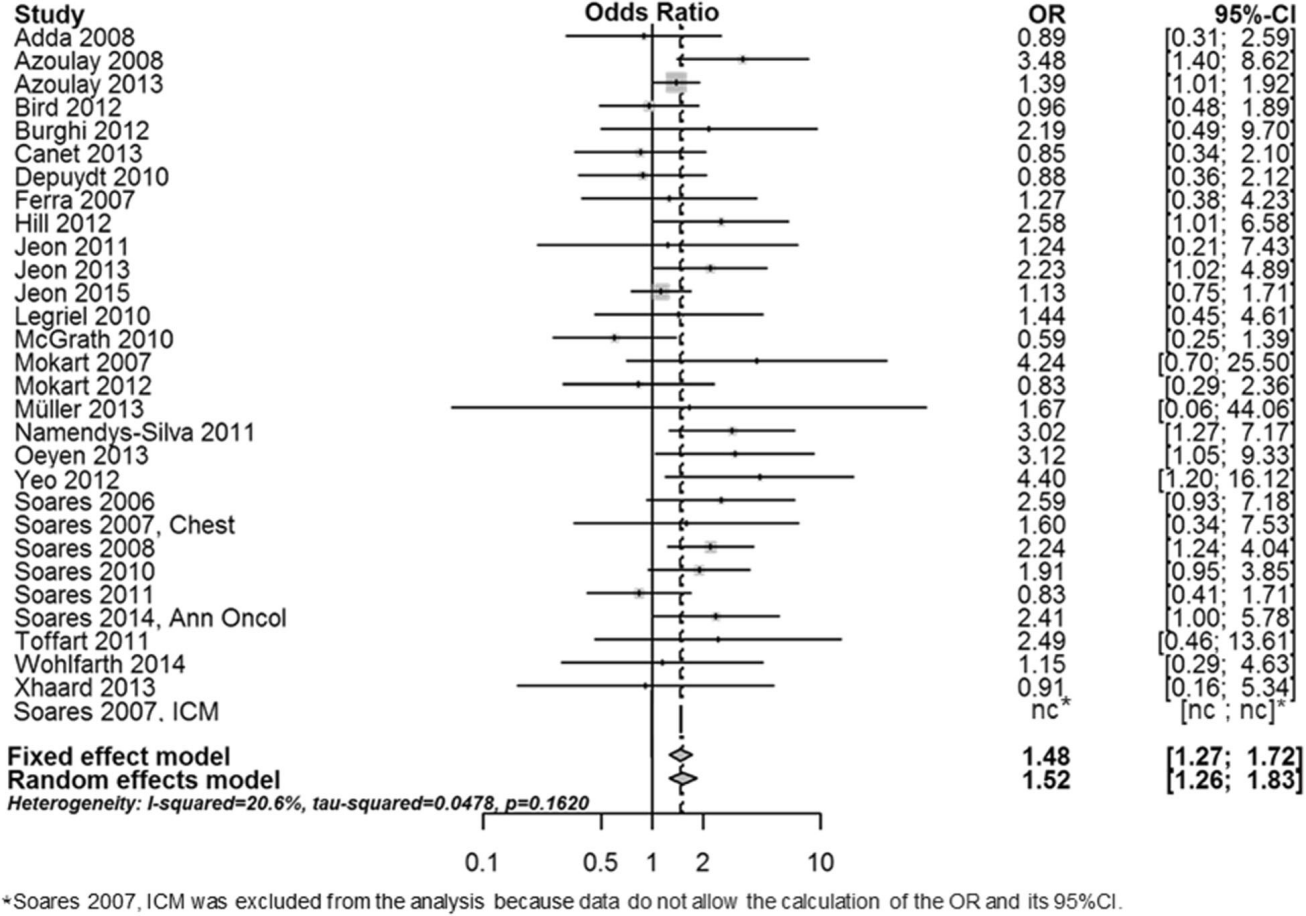
Summary of adjusted prognostic impact of neutropenia on mortality in the included studies. CI confidence interval, OR odds ratio, ICM Intensive Care Med, n.c., not counted (no event)

After adjustment for confounders, and taking study effect into account, neutropenia was independently associated with mortality (OR 1.41; 95% CI 1.23–1.62; *P* = 0.03) (Table 3, Fig. 2).

**Table 3:** Factors independently associated with mortality after adjustment for confounders (mix-linear model taking study effect into account)

Variable	Odds ratio	95% CI	*P* value
Neutropenia	1.41	1.23–1.62	0.03
Age < 50 years	Reference	–	–
Age 50–59 years	1.11	0.95–1.28	0.18
Age 60–69 years	1.32	1.13–1.53	< 0.001
Age 69+ years	1.66	1.43–1.94	< 0.001
Solid tumors (vs HM)	0.69	0.58–0.81	< 0.001
Allogeneic HSCT	1.91	1.50–2.43	< 0.001
Mechanical ventilation	3.01	2.66–3.41	< 0.001
Vasopressors	2.07	1.83–2.35	< 0.001
Renal replacement therapy	1.50	1.29–1.75	< 0.001

*CI* confidence interval, *HM* hematological malignancy, *HSCT* hematopoietic stem cell transplantation

### Influence of neutropenia in predefined subgroups

When analyzed separately, neither admission period, underlying malignancy nor need for mechanical ventilation modified the influence of neutropenia on outcome (Additional file 1: Tables S1–S3).

In patients admitted after 2007 (median admission period; *n* = 3563, including 880 neutropenic patients), neutropenia was independently associated with an increased mortality (OR 1.40; 95% CI 1.16–1.70; *P* < 0.001).

In patients with hematological malignancy (*n* = 4149, including 1470 neutropenic patients), neutropenia was independently associated with an increased mortality (OR 1.30; 95% CI 1.11–1.51; *P* < 0.001).

Among the included patients, data on use of G-CSF was available for 1949 patients (25.9%). Among the 788 patients receiving G-CSF, 587 were neutropenic. After adjustment for confounders (Additional file 1: Table S4), neutropenia was not independently associated with outcome (OR 1.03; 95% CI 0.70–1.51; *P* = 0.90).

## Discussion

This large dataset resulting from systematic review of individual data confirms neutropenia to be independently associated with mortality in critically ill cancer patients. According to our results, the prognostic impact of neutropenia was unchanged when stratifying for malignancy, period of ICU admission or use of mechanical ventilation. However, in patients treated with G-CSF, neutropenia was no longer associated with mortality, suggesting that the use of G-CSF may influence the prognostic impact of neutropenia in this setting.

Neutropenia remains an accepted side effect of most treatments administered to hematological patients [52]. Neutropenia is associated with complications that include severe sepsis [53], acute respiratory failure [54] and specific conditions such as neutropenic enterocolitis [55]. Although these side effects are likely to influence the outcome of critically ill patients, results of studies in this field remain controversial. Although neutropenia remains associated with a poor outcome in general ICU populations [16], several recent studies failed to demonstrate an impact of neutropenia on the outcome of critically ill cancer patients [8, 12]. The numerous mechanisms of immune deficiency in these patients, along with the prognostic influence of disease severity or need for organ support therapies, might explain these negative findings. A previous systematic review, based on aggregated data, suggested that neutropenia was associated with a 10% increase in overall mortality but the result may have been confounded by studies with negative findings due to lack of statistical power [17]. In fact, the influence of neutropenia was no longer significant after adjusting for confounders but in a limited study population [17]. Results of our study confirm that, even after adjustment for confounders, neutropenia is associated with a poor outcome. These data strongly suggest that neutropenia, conversely to the recently published recommendations [15], should be considered a prognostic factor. Additional studies are needed to confirm our results and to identify room for improvements in the management of this specific population.

In most of the predefined subgroups, namely according to underlying malignancy, use of mechanical ventilation or according to ICU admission period, the impact of neutropenia on outcome was unchanged. However, it must be noted that the impact of neutropenia was no longer associated with outcome in patients treated with G-CSF. Prophylactic use of G-CSF in patients with hematological malignancy or solid tumors has proven efficacy in decreasing the risk or duration of neutropenia, in limiting the risk of infectious disease and in specific settings decreasing both overall mortality and infection-related mortality [56, 57]. Conversely, use of G-CSF in patients with already overt infections (curative G-CSF) was found to have a limited benefit in neutropenic patients [58, 59]. Data regarding interest of prophylactic or curative use of G-CSF are limited to studies with high risk of bias and suggest a limited efficacy in this setting [60–62]. In addition, G-CSF remains associated with potential side effects, including risk of worsening respiratory status during neutropenia recovery [63]. Our study is the first to date to suggest indirectly that G-CSF may limit the prognostic impact of neutropenia in critically ill patients. Although this result is insufficient to modify existing recommendations, additional interventional studies in this setting may be warranted.

This study has several important limitations. Firstly, despite the biological plausibility, this study at best demonstrated a statistical association between neutropenia and mortality. Whether this statistical association may be due to a causal relationship remains to be demonstrated. Secondly, the observed dependent association that might have been affected by allocation bias not taken into account by our analysis. In line with this, available data did not allow adjustment for center or volume effect, assessment of the impact of organizational processes [64], influence of duration of neutropenia or influence of several management strategies including impact of antifungal prophylaxis. Last, only a quarter of identified studies were ultimately included. However, it must be noted that the large dataset, and the analysis adjusting for study influence and modeling of unrecorded data by using a mixed effect model, should have, at least partly, taken these effects into account. In line with this, several arbitrary choices were made during analysis as regards the study inclusion period or cutoff point to define the ICU admission period. The lack of other reliable cutoff points, however, is to be noted when taking into account these limits. Last, the influence of G-CSF was assessed only indirectly, in a subset of patients in whom the use of G-CSF was recorded, without information regarding the rational for its prescription. Thus, our negative results might reflect either a lack of statistical power or an inclusion bias. Nevertheless, our results are a strong plea for further interventional studies to assess the influence of G-CSF in critically ill patients with neutropenia.

## Conclusion

This systematic review, comprising 7515 patients, suggests a meaningful survival in neutropenic critically ill patients despite an independent association with poor outcome. Neither underlying malignancy, a period of admission nor use of mechanical ventilation significantly modified this result. Interestingly, neutropenia was no longer significantly associated with outcome in patients treated with G-CSF. Thus, our results may suggest a beneficial effect of G-CSF in critically ill cancer patients and serve as a plea for additional studies in this field.

## Key messages

- This systematic review of individual data suggests a meaningful survival in neutropenic critically ill patients despite an independent association with poor outcome.
- Neutropenia is no longer significantly associated with outcome in patients treated with G-CSF, suggesting a beneficial effect of this therapy in critically ill cancer patients.
- The independent association of neutropenia with poor outcome suggest additional research to be required in way to limit excess of mortality in this subgroup of critically-ill patients.
- Potential beneficial effect of G-CSF in critically-ill neutropenic patients is a plea for additional studies assessing benefits of therapeutic G-CSF in this subgroup of patients.

## Additional file

Additional file 1: **Table S1.** Factors independently associated with mortality after adjustment for confounders among patients with ICU admission after 2007 (mix-linear model taking study effect into account). **Table S2.** Factors independently associated with mortality after adjustment for confounders among patients with hematological malignancy (mix-linear model taking study effect into account). **Table S3.** Factors independently associated with mortality after adjustment for confounders among patients requiring mechanical ventilation (mix-linear model taking study effect into account). **Table S4.** Factors independently associated with mortality after adjustment for confounders among patients receiving G-CSF (mix-linear model taking study effect into account) (DOC 58 kb)

## Abbreviations

APACHE: Acute Physiology and Chronic Health Evaluation
G-CSF: Granulocyte colony stimulating factor
HSCT: Hematopoietic stem cell transplant
ICU: Intensive care unit
SAPS: Simplified Acute Physiology Score
