# Supplementary material for: Influence of neutropenia on mortality of critically ill cancer patients: results of a meta-analysis on individual data
Source: Crit Care. 2018 Dec 4;22:326. doi: 10.1186/s13054-018-2076-z (PMC6280476; doi:10.1186/s13054-018-2076-z)
Supplement: Supplementary file 1 — Table S1. Factors independently associated with mortality after adjustment for confounders among patients with ICU admission after 2007 (mix-linear model taking study effect into account). Table S2. Factors independently associated with mortality after adjustment for confounders among patients with hematological malignancy (mix-linear model taking study effect into account). Table S3. Factors independently associated with mortality after adjustment for confounders among patients requiring mechanical ventilation (mix-linear model taking study effect into account). Table S4. Factors independently associated with mortality after adjustment for confounders among patients receiving G-CSF (mix-linear model taking study effect into account) (DOC 58 kb) [file 13054_2018_2076_MOESM1_ESM.doc]

**Supplementary appendix**

**Influence of neutropenia on mortality of critically ill cancer patients: Results of a systematic review on individual data.**

Quentin GEORGES et al.

**Table S1. Factors independently associated with mortality after adjustment for confounders among patients with ICU admission after 2007 (mix-linear model taking study effect into account)**

|  | **Odds ratio** | **95% CI** | ***P* value** |
| --- | --- | --- | --- |
|  |  |  |  |
| Neutropenia | 1.40 | 1.16-1.70 | <0.001 |
| Age < 50 years  Age 50-59  Age 60-69  Age 69+ | Ref  1.09  1.33  1.69 | -  0.88-1.35  1.07-1.67  1.34-2.13 | -  0.45  0.01  <0.001 |
| Solid tumors (vs. HM) | 0.74 | 0.57-0.96 | 0.02 |
| Allogeneic HSCT | 2.26 | 1.68-3.05 | <0.001 |
| Mechanical ventilation | 2.20 | 1.68-2.65 | <0.001 |
| Vasopressors | 2.10 | 1.74-2.53 | <0.001 |
| Renal Replacement Therapy | 2.17 | 1.75-2.69 | <0.001 |

CI: confidence interval; HM: Hematological malignancy; HSCT: Hematopoietic Stem Cell Transplantation

**Table S2. Factors independently associated with mortality after adjustment for confounders among patients with hematological malignancy (mix-linear model taking study effect into account)**

|  | **Odds ratio** | **95% CI** | ***P* value** |
| --- | --- | --- | --- |
|  |  |  |  |
| Neutropenia | 1.30 | 1.11-1.51 | <0.001 |
| Age < 50 years  Age 50-59  Age 60-69  Age 69+ | Ref  1.15  1.39  1.75 | -  0.95-1.39  1.14-1.69  1.43-2.17 | -  0.15  <0.001  <0.001 |
| Allogeneic HSCT | 1.91 | 1.50-2.45 | <0.001 |
| Mechanical ventilation | 2.55 | 2.16-3.03 | <0.001 |
| Vasopressors | 2.21 | 1.87-2.60 | <0.001 |
| Renal Replacement Therapy | 1.47 | 1.24-1.76 | <0.001 |

CI: confidence interval; HM: Hematological malignancy; HSCT: Hematopoietic Stem Cell Transplantation

**Table S3. Factors independently associated with mortality after adjustment for confounders among patients requiring mechanical ventilation (mix-linear model taking study effect into account)**

|  | **Odds ratio** | **95% CI** | ***P* value** |
| --- | --- | --- | --- |
|  |  |  |  |
| Neutropenia | 1.69 | 1.39-2.06 | <0.001 |
| Age < 50 years  Age 50-59  Age 60-69  Age 69+ | Ref  1.06  1.22  1.76 | -  0.87-1.29  0.99-1.49  1.42-2.18 | -  0.57  0.06  <0.001 |
| Solid tumors (vs. HM) | 0.73 | 0.59-0.90 | 0.01 |
| Allogeneic HSCT | 1.71 | 1.18-2.52 | 0.01 |
| Vasopressors | 2.40 | 2.03-2.83 | <0.001 |
| Renal Replacement Therapy | 1.44 | 1.17-1.77 | <0.001 |

CI: confidence interval; HM: Hematological malignancy; HSCT: Hematopoietic Stem Cell Transplantation

**Table S4. Factors independently associated with mortality after adjustment for confounders among patients receiving G-CSF (mix-linear model taking study effect into account)**

|  | **Odds ratio** | **95% CI** | ***P* value** |
| --- | --- | --- | --- |
|  |  |  |  |
| Neutropenia | 1.03 | 0.70-1.51 | 0.90 |
| Allogeneic HSCT | 1.78 | 1.13-2.86 | 0.01 |
| Mechanical ventilation | 2.19 | 1.42-3.39 | <0.001 |
| Vasopressors | 1.26 | 0.81-1.96 | 0.30 |
| Renal Replacement Therapy | 1.50 | 0.99-2.28 | 0.06 |

CI: confidence interval; HM: Hematological malignancy; HSCT: Hematopoietic Stem Cell Transplantation ; G-CSF: granulocyte colony stimulating factors
